# Patterns of diagnostic marker assessment in adult diffuse glioma: a survey of the European Confederation of Neuropathological Societies (Euro-CNS) 

**DOI:** 10.5414/NP301009

**Published:** 2016-12-14

**Authors:** Adelheid Woehrer, Bjarne W. Kristensen, Anne Vital, Johannes A. Hainfellner

**Affiliations:** 1 Institute of Neurology and Comprehensive Cancer Center CNS Unit, Medical University of Vienna, Austria,; 2 Department of Pathology, Odense University Hospital and Institute of Clinical Research, University of Southern Denmark, Denmark, and; 3 Institute of Neuroscience, Centre Hospitalier Universitaire de Bordeaux, Bordeaux, France

**Keywords:** diffuse glioma, molecular markers, integrated diagnosis, neuropathology survey

## Abstract

The 2016 update of the WHO classification has introduced an integrated diagnostic approach that incorporates both tumor morphology and molecular information. This conceptual change has far-reaching implications, especially for neuropathologists who are in the forefront of translating molecular markers to routine diagnostic use. Adult diffuse glioma is a prototypic example for a group of tumors that underwent substantial regrouping, and it represents a major workload for surgical neuropathologists. Hence, we conducted a survey among members of the European Confederation of Neuropathological Societies (Euro-CNS) in order to assess 1) the extent to which molecular markers have already been incorporated in glioma diagnoses, 2) which molecular techniques are in daily use, and 3) to set a baseline for future surveys in this field. Based on 130 responses from participants across 40 nations neuropathologists uniformly rate molecular marker testing as highly relevant and already incorporate molecular information in their diagnostic assessments. At the same time however, the survey documents substantial differences in access to crucial biomarkers and molecular techniques across geographic regions and within individual countries. Concerns are raised concerning the validity of test assays with *MGMT*, 1p19q, and ATRX; being perceived as most problematic. Neuropathologists advocate the need for international harmonization of standards and consensus guidelines, and the majority is willing to actively engage in interlaboratory trials aiming at quality control (Figure 1).

## Context 

The 2016 update of the WHO classification of tumors of the central nervous system has introduced an integrated diagnostic approach that incorporates both tumor morphology and molecular information [2]. This conceptual change has fundamental and far-reaching implications, especially for neuropathologists who are in the forefront of translating molecular findings in large-scale cohort studies to the diagnostic setting of individual patients. Hence, neuropathologists find themselves continuously confronted with the set-up of additional molecular tests as well as the interpretation, validation, and integration of test results. While the proposed integration of molecular markers aims at harmonizing diagnostic standards, it introduces complexity pertaining to technical, personnel, time, and cost-related issues. 

Adult diffuse glioma comprises the most common group of primary brain tumors and thus, represents a major workload for surgical neuropathologists. It is a prototypic example of a group of tumors that underwent substantial regrouping based on molecular constellations [3]. Molecular changes such as *IDH* mutations, 1p19q codeletion, and *ATRX* mutations are meanwhile considered prerequisites for precise subtyping into diffuse astrocytoma, oligodendroglioma, and glioblastoma categories [3, 4, 5]. Likewise, *TERT* promoter mutations have been implicated as relevant prognostic factors [5]. Regarding glioblastoma, the *MGMT* promoter methylation status further impacts therapeutic choices in elderly patients [6]. All these markers can be assessed on routinely processed formalin-fixed and paraffin-embedded tumor tissues. Two molecular alterations, namely the *IDH1 R132H* mutation and *ATRX* mutation with subsequent loss of ATRX protein expression, are mainly evaluated by immunohistochemistry [7]. All other markers require further molecular techniques such as targeted gene sequencing for *IDH2,* rare *IDH1,* and *TERT* mutations, as well as fluorescence in situ hybridization (FISH) or whole chromosomal arm spanning multiplex ligation-dependent probe amplification (MLPA) for 1p19q codeletion testing [8]. Likewise, the *MGMT* promoter methylation status is commonly evaluated using pyrosequencing or methylation-specific polymerase chain reaction (MSP) [9, 10]. More advanced methods that allow for simultaneous assessment of multiple markers, e.g., brain tumor-associated gene panel sequencing, exome sequencing, or DNA methylation profiling are trending but their availability is still limited to specialized laboratories [11, 12]. 

## Objectives 

We herein sought to document current practice patterns in the molecular assessment of adult diffuse glioma during a period of transition after the revised WHO classification was released earlier in 2016. We specifically sought to: 1) assess the extent to which the different molecular markers have already been incorporated into routine practice, 2) assess which molecular techniques and platforms are in daily use or will be implemented in the near future, and 3) to set a baseline evaluation for future surveys in this area. 

## Strategy for question design, collection and summary of responses 

The instrument of choice for this study was a survey targeting neuropathologists engaged in brain tumor diagnostics using the European Confederation of Neuropathological Societies (Euro-CNS) as communication platform. Euro-CNS aims at promoting and maintaining the harmonization of neuropathological training and practice across Europe (http://www.euro-cns.org). It comprises 21 national organizations with over 1,000 individual memberships. The survey included questions pertaining to the overall significance of molecular marker testing, its impact on diagnostic evaluations, current and near future availability of molecular platforms, and relevance of quality control such as ring trials and consensus guidelines. Participants were asked to provide information on current place of work. Most questions offered multiple choices with a broad range of answers presented in random order. The survey was designed by the investigators and reviewed by members of the Euro-CNS executive team prior to its release in paper- and web-based format on occasion of the 11^th^ European Congress of Neuropathology (ECNP) in Bordeaux, France, July 6 – 9, 2016. In addition to the congress participants (294 registrations), all Euro-CNS members were invited via email to complete the survey online. It was designed and tested to allow a completion time of ~ 5 minutes. Responses were anonymously collected and stored online using Google spreadsheet. The hyperlink to the online survey was accessible through October 17, 2016. Summary statistics were prepared for responses to each question and presented as percentage or absolute number of responses. Test statistics included χ^2^-test for categorical variables and t-test for numeric values. All statistical analyses and graphic representations were performed with Microsoft Excel v14.4.8, SPSS-statistics v23, and MATLAB R2015b. The World 2016 ranking of gross domestic products (GDP) per capita was used to stratify countries into high- (top 20% GDP per capita) vs. lower-income countries (lower 80% GDP per capita). Subgroup analysis was performed for countries with more than 10 respondents, respectively (top-responding countries). Detailed information on the number of practicing neuropathologists actively engaged in brain tumor diagnostics per country was not available. 

## Results 

A total of 130 participants completed the survey. 45 responses were collected at the occasion of ECNP 2016 and another 85 respondents followed the invitation by email. The vast majority of respondents (122/130, 93.8%) indicated neuropathology or general pathology with sub-specialization in neuropathology as their profession. In contrast, respondents from other disciplines were rare and included molecular biologists (4 respondents), neurologists (3 respondents), or neurosurgeons (1 respondent). Overall, responses were collected from 40 different countries across all geographic regions with 75% of respondents indicating to work in Europe followed by North America (11%) and Asia (9%) (Figure 2). Thus, 76.9% of respondents work in high-income countries (top 20% GDP per capita) with an overrepresentation of European countries. Top-responding countries comprised Germany with 21 respondents (16.2%), followed by the United Kingdom with 18 respondents (13.8%), and Spain and the United States of America with 11 respondents each (8.5%). In contrast, single respondents were on record from 17 different countries (17/40, 42.5%) contributing 13.1% of total responses. 

## Responses to questions 1 – 10 

The overwhelming majority of neuropathologists considers molecular testing as important or very important (93.6%) and already incorporates molecular information to some extent in their routine diagnostic assessments (96.0%) (Question 1, 2). Among the widely implemented markers are *IDH1* (93.1%), 1p19q (85.4%), and *MGMT* (70.0%), whereas *TERT* (17.7%) is the least widely evaluated marker (Question 4a, Question 4b Question 4c). 64% of respondents routinely assess 4 or more of the 6 proposed markers whereas three participants report to have no access to any marker (2.3%). Two out of these 3 respondents rate the overall relevance of marker testing as low. Laboratories that routinely evaluate 1 or 2 markers typically focus on *IDH1* and 1p19q. Those, who extend their marker panel to a third often include ATRX or *MGMT*. Respondents from lower income countries assess on average fewer markers as compared with those from high-income countries (2.5 vs. 4.3 markers, p < 0.0005). However, substantial within-country heterogeneity in the number and combination of markers is observed when restricting the analysis to top-responding countries only, which collectively fall in the high-income category (Question 4c). 50% of neuropathologists indicate to no longer use “oligoastrocytoma” as histological diagnosis (Question 3). Those are more likely to report integrated diagnostic use of molecular profiles (p = 0.005) and are more likely to have access to relevant markers, i.e., *IDH1*, 1p19q, and ATRX (p = 0.013). 

FISH is by far the most broadly available technique (71.5%), followed by gene panel sequencing (46.2%), and MSP (45.4%) (Question 5a, Question 5b, Question 5c). In contrast, only 10% of respondents report the routine use of methylation arrays. The latter include few respondents from Europe and North America as well as a single respondent from South Africa. Overall, roughly 80% of all laboratories use 2 or more molecular platforms, 53.8% 3 or more. In contrast, 6.9% of respondents indicate to have no access to any molecular technique. The combinations of the individual techniques that are used vary substantially across laboratories with lower-income countries having access to fewer techniques (1.8 vs. 2.9 techniques, p < 0.0005). Again, within-country heterogeneity is prominent upon subgroup analysis (Question 5c). Considering near future availability of additional techniques, 60.0% of neuropathologists aim at implementing 1 additional technique, another 19.2% 2 or more techniques (Question 6a). In contrast, 20.8% of respondents state to have no concrete plans with free-text comments suggesting either limited resources or already broad access to molecular platforms. Subgroup analysis for high- vs. lower-income countries yields similar trends with somewhat divergent ratios for future use of MLPA, next-generation sequencing, and DNA methylation arrays that are more heavily demanded in high-income countries (Question 6b). 58% of neuropathologists are concerned about the analytical test performance of any molecular marker with *MGMT* (20 nominations), 1p19q (16 nominations), and ATRX (15 nominations); they considered most problematic (Question 7, Question 8). Concerns about the analytical test performance are not associated with self-reported relevance scores of molecular marker testing (p = 0.42). 90% of neuropathologists support the notion that there is a need for international guidelines on marker evaluation and the majority is willing to participate in ring trials (87.7%) (Question 9, Question 10). 

## Summary & key messages 

The present Euro-CNS survey documents practice patterns pertaining to the molecular classification of adult diffuse glioma. It stands as one of the first efforts that specifically focus on neuropathologists who are responsible for implementing the new “integrated” diagnostic format as advocated by the updated WHO classification. Even though our survey has a strong focus on the European neuropathology community and thus might not be fully representative for the international scenario, it provides useful insights and complements previous surveys in this field [13, 14]. 

### Neuropathologists rate molecular marker testing as highly relevant and already incorporate molecular information in their glioma diagnoses 

Overwhelmingly positive responses underscore the broad support among neuropathologists towards the integration of molecular markers in their daily routine. This positive attitude is in line with a previous questionnaire among members of the Society of Neuro-Oncology [14]. In their survey, however, the authors noted less enthusiasm among neuropathologists as compared with other disciplines such as oncologists or neurosurgeons. As potential reason they suggested relatively stronger concerns among neuropathologists who are familiar with the challenges of implementing new methods and their practical hurdles; a conclusion, which seems perfectly true in the light of the results of the current survey. 

### Diagnostic marker assessment varies across institutions and geographic regions 

The present survey focused on a set of molecular markers that are meanwhile considered crucial in the assessment of adult diffuse glioma. Among the proposed markers, *IDH1*, 1p19q, and *MGMT* are probably the best-established ones and their diagnostic, prognostic, and predictive relevance has been demonstrated and confirmed in several independent studies [15, 16, 17]. Accordingly, the majority of neuropathologists have already successfully incorporated them in their routine diagnostic evaluations. Nevertheless, a substantial fraction of neuropathologists reports to have no access to 1 or more of these top 3 markers with *MGMT* being the least available. At the same time, *MGMT* testing is perceived most problematic, which points towards substantial technical issues that prevent its smooth translation into routine use. Similar reasons might account for the less prevalent use of *IDH2* testing, which – in contrast to the most common *IDH1* R132H mutation – definitely requires a sequencing-based approach. Among the various markers, *TERT* mutation is the most recently implicated one and not explicitly included in the WHO classification [5]. Thus, its restricted use in daily routine comes as no surprise. Overall, practice patterns vary substantially not only across geographic regions but also within individual countries suggesting considerable regional differences in terms of available resources and training (see also “Access to molecular techniques varies across institutions and geographic regions” of molecular techniques). 

### Access to molecular techniques varies across institutions and geographic regions 

The main purpose of the WHO tumor classification is to provide a consensus classification for measuring cancer burden in a standardized way worldwide. It is not the task of WHO to provide guidelines on which molecular tests to use. Accordingly, the updated WHO brain tumor classification provides no recommendations pertaining to which molecular techniques to choose. As recognized international or worldwide guidelines do not exist up to now, there is ample freedom left to the individual centers. This may pose certain problems as different techniques are associated with inherent advantages and disadvantages and thus, leave room for inter-laboratory differences. 

The most widely available molecular technique and seemingly standard in (neuro)pathology laboratories is FISH, a cost-effective but laborious and time-intense method that offers the advantage of morphology-based evaluations, which per se are intuitive to pathologists. On the other end of the spectrum is array-based DNA methylation profiling, which is available to only very few neuropathologists mostly based in Europe and the United States (multiple respondents per institution were allowed and single respondents might have included DNA methylation arrays in their portfolio as they refer cases to a tertiary center). 

Overall, more than half of all neuropathologists report the routine use of 3 or more molecular platforms. The combination of techniques, however, varies substantially without emerging “optimal” technical set-up. Hence, some observations deserve to be mentioned. 1) A substantial number of neuropathologists who routinely use panel-sequencing report to no longer perform targeted gene sequencing. 2) Similar mutual trends are observed for MSP vs. pyro-sequencing and FISH vs. MLPA with clear preferences for MSP and FISH, respectively. 3) Most importantly, while some institutions have access to almost all platforms, a considerable number of neuropathologists report to have no or only limited access to molecular techniques, thus, indicating substantial inequalities of diagnostic standards across geographic regions and institutions. This is well in line with a previous survey of the International Society of Neuropathology that demonstrated huge differences in access to molecular techniques – not only on a global scale but also within Europe [13]. These discrepancies reflect differences in healthcare systems with generally weaker performance of economically weaker countries. However, within-country heterogeneity emerges as a further contributing factor, which together with the high number of single-respondents per country points towards centralization of diagnostic services in only few specialized centers per country. This trend is likely to further increase with the increasing complexity of diagnostic standards. Thus, for smaller centers with fewer case numbers it might be out of scope to invest in additional molecular tests; instead, they might choose to outsource diagnostic services. On a national (and maybe even international) scale this centralization will lead to a considerable restructuring of existing infrastructures and resources, and attention will need to be paid to warrant equal, nationwide access to diagnostic services. Nevertheless, when asked about near-future plans pertaining to additional molecular techniques the majority of respondents nominate one or more techniques including targeted/panel gene sequencing and DNA methylation arrays. Especially the incorporation of high-throughput screening platforms for routine diagnostic purposes constitutes a rapidly evolving theme in neuropathology as in general pathology [18]. In that sense, cancer-associated gene panel sequencing seems to strike a balance between cost-effective screening for diagnostically useful/actionable mutations and reduced data analysis burden as compared with other genome-wide platforms such as DNA methylation profiling or whole exome sequencing [11, 19, 20]. Of note, the present survey did not specifically address whether gene panel sequencing is performed in-house or in cooperation with emerging biotech companies such as FoundationOne^®^ (Cambridge, MA, USA) [21] or IBM Watson for Genomics/Quest Diagnostics™ (New York, NY, USA) – which would have added another interesting aspect. 

Over the last years, DNA methylation profiling has emerged as a particularly appealing candidate technique for brain tumor diagnostics as it 1) allows for the simultaneous assessment of multiple markers (e.g., global DNA methylation levels as surrogate for *IDH* mutations, methylation status of the *MGMT* gene promoter, and copy numbers of chromosomal arms 1p19q) and 2) makes use of epigenetic signatures as tracers for tissues/cells of origin, which might aid in diagnostically challenging cases [12]. Still, for the moment DNA methylation profiling is associated with considerable costs and requires substantial input from bioinformatics. Thus, not surprising its routine use is restricted to single centers. Similarly, first neuropathologists indicate to have concrete plans to implement exome sequencing, which was per se not included in the predefined set of molecular techniques surveyed. In line with DNA methylation profiling, exome sequencing requires substantial input from bioinformatics with all related issues such as the standardization of computational pipelines, quality control of data acquisition and storage, as well as prolonged time to diagnosis. Moreover, genetic patient counseling including the eventual reporting of incidental findings emerges as new but important challenge, which would ideally be addressed in a patient-oriented, non-profit medical setting [18, 22]. Likewise, despite ever falling prices it is currently unclear whether publicly funded healthcare will be able to cover related financial costs. Hence, it will be interesting to follow the various public/private developments and the increasing use of these advanced platforms across different countries and geographic regions. 

### Neuropathologists advocate consensus recommendations on marker testing 

More than half of all neuropathologists are concerned about the analytical test performance of any molecular marker and when asked to nominate the most problematic, interestingly, the longest-implicated ones such as *MGMT* and 1p19q pop up. While clinicians are enthusiastic about introducing biomarkers early in clinics and as stratification factors in trials, the thorough validation of test assays is laborious and requires compliance with governmental authorities and regulations including formalized in-house validation procedures in large centers, the European Union’s CE marking and IVD procedure, or the CLIA regulations and CAP guidelines in the US. Nevertheless, the herein expressed concerns add a cautious note that even for long-standing markers and certified tests there is room for further improving quality control and harmonization of international standards. The third most commonly implicated marker was ATRX, which exemplifies that even straightforward techniques such as immunohistochemistry can be tricky and some antibodies might be associated with inconsistent staining results when using different automated staining systems. Overall, such practical feedback is critically needed. With regard to Europe and beyond, an international professional body such as Euro-CNS could substantially contribute to the harmonization of standards and improvement of diagnostic quality by means of international scientific interlaboratory comparisons. In this sense activities such as ring trials seem greatly welcomed by the majority of neuropathologists. 

Our survey has several drawbacks, most prominently including a selection bias towards European respondents given the Euro-CNS platform as chosen approach. Thus, our results do not fully capture practice patterns on a global level. Wide parts of Southern America, Africa, and Asia are left open and more focused efforts would need to be undertaken to cover those underrepresented populations. However, even among Euro-CNS members the overall response rate was relatively low, which might be due to several reasons. 1) Many neuropathologists have a different focus apart from brain tumors such as neurodegenerative or neuromuscular diseases. 2) Neuropathologists with limited available resources and frank problems to implement new markers might be less motivated to participate in a targeted survey. 3) Ultimately, it is difficult to estimate the maximum feasible target population, i.e., currently practicing neuropathologists per country as Euro-CNS members comprise various disciplines including basic neuroscientists and related clinical disciplines. 

The survey was designed to provide summary views on molecular markers and access to molecular techniques, rather than enabling detailed linkage of the individual markers with their corresponding techniques. That, however, would be of interest and future surveys would benefit from including this information. Similarly, it would be reasonable to extend this approach to other brain tumor entities and additional markers. 

In summary, the current Euro-CNS survey demonstrates broad support of the neuropathology community towards the integration of molecular markers in routine diagnostic assessments as advocated by the updated WHO classification. Despite enthusiasm, access to molecular markers and techniques varies substantially across geographic regions and within individual countries. In addition, major concerns are raised pertaining to the validity of test assays with *MGMT*, 1p19q, and ATRX being perceived as most problematic. Uniformly, neuropathologists advocate the need for international consensus guidelines and the majority is willing to actively engage in quality control, harmonization of standards, and interlaboratory comparisons by means of ring trials. 

## Acknowledgments 

We appreciate the time investment and active participation of all survey respondents. We further thank the Euro CNS executive team and ECNP 2016 congress officials for providing the framework and Ilja Huang, Euro-CNS Administrator, for excellent technical support. We acknowledge Dr. Bernhard Baumann, Center for Medical Physics and Biomedical Engineering, Medical University of Vienna, Austria, for assistance with graphic data representation and Dr. David N. Louis, Chair of Massachusetts General Pathology and Harvard Medical School, for constructive discussion and valuable feedback. 

## Conflict of interest 

The authors declare no conflict of interest. 

**Figure 1.**
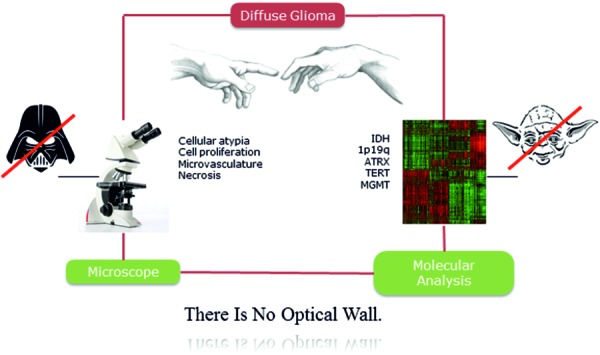
Graphical abstract in response to Ramaswamy and Taylor, Cancer Cell 2016 [1].

**Figure 2.**
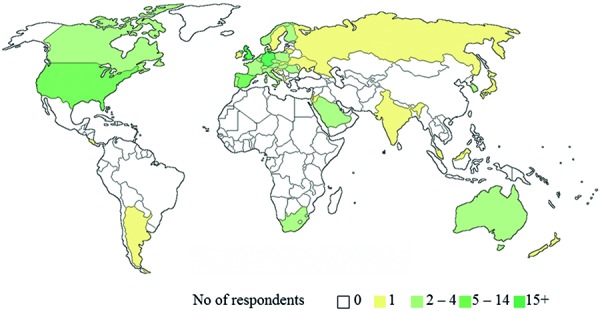
Place of work.

**Question 1 – 3.**
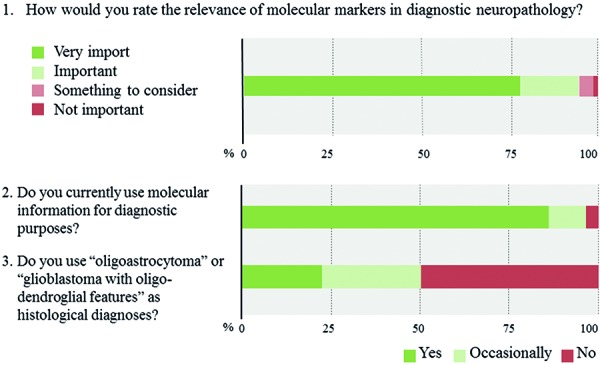
Responses to questions 1 – 3.

**Question 4a.**
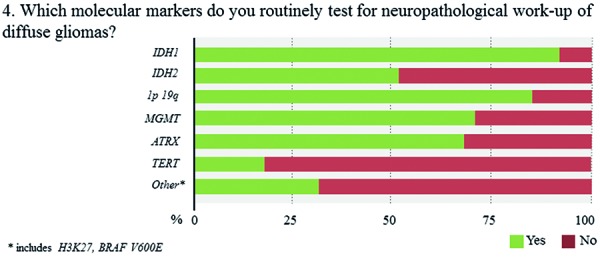
Response to question 4.

**Question 4b.**
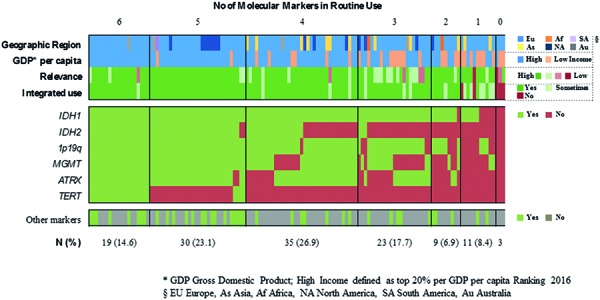
Response to question 4.

**Question 4c.**
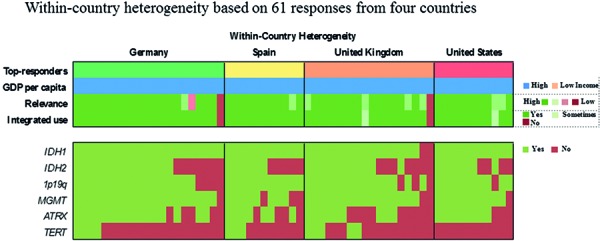
Response to question 4.

**Question 5a.**
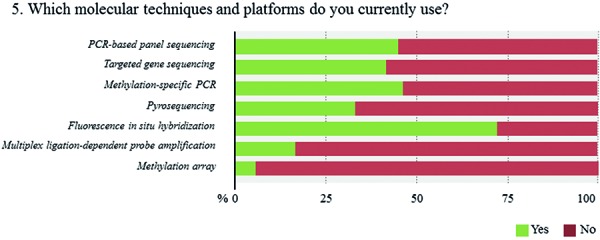
Response to question 5.

**Question 5b.**
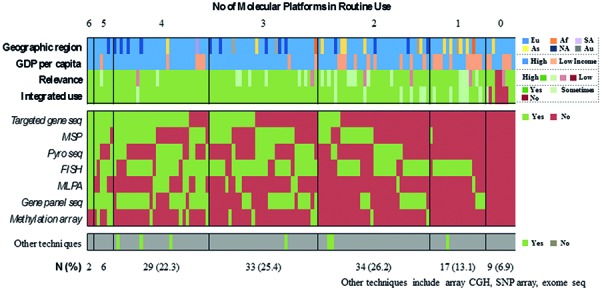
Response to question 5.

**Question 5c.**
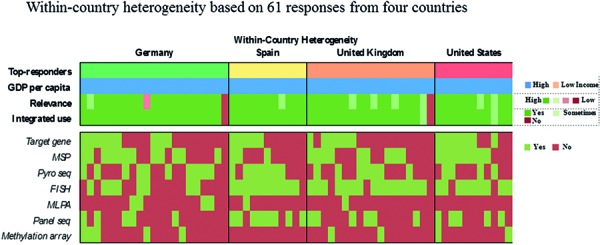
Response to question 5.

**Question 6a.**
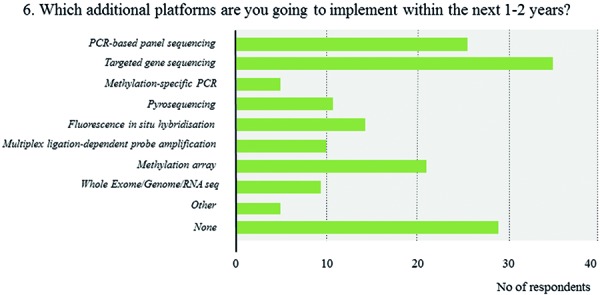
Response to question 6.

**Question 6b.**
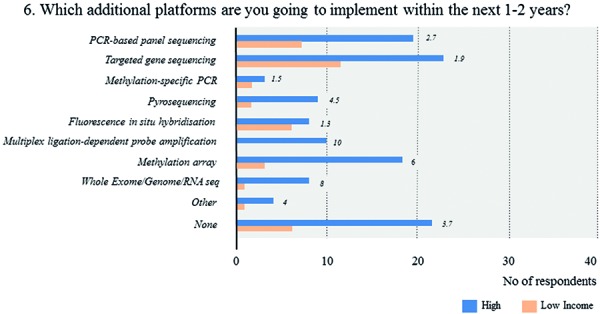
Response to question 6.

**Question 7 – 8.**
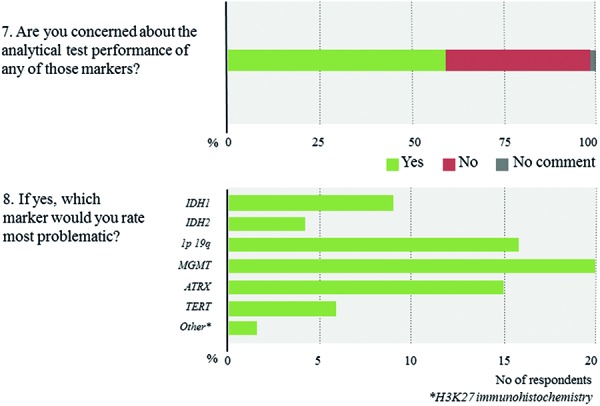
Responses to questions 7 and 8.

**Question 9 – 10.**
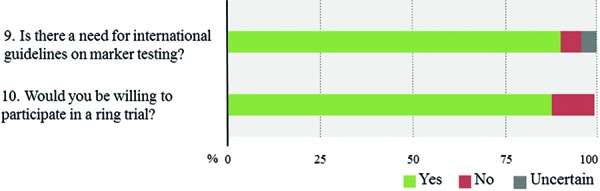
Responses to questions 9 and 10.

## References

[1] Ramaswamy V Taylor MD Fall of the Optical Wall: Freedom from the Tyranny of the Microscope Improves Glioma Risk Stratification. Cancer Cell. 2016; 29: 137–138. 26859451 10.1016/j.ccell.2016.01.009

[2] Louis DN, Ohgaki H Wiestler OD Cavenee WK World Health Organization Histological Classification of Tumours of the Central Nervous System. France: International Agency for Research on Cancer; 2016.

[3] Ceccarelli M Barthel FP Malta TM Sabedot TS Salama SR Murray BA Morozova O Newton Y Radenbaugh A Pagnotta SM Anjum S Wang J Manyam G Zoppoli P Ling S Rao AA Grifford M Cherniack AD Zhang H Poisson L Molecular Profiling Reveals Biologically Discrete Subsets and Pathways of Progression in Diffuse Glioma. Cell. 2016; 164: 550–563. 26824661 10.1016/j.cell.2015.12.028 PMC4754110

[4] Brat DJ Verhaak RG Aldape KD Yung WK Salama SR Cooper LA Rheinbay E Miller CR Vitucci M Morozova O Robertson AG Noushmehr H Laird PW Cherniack AD Akbani R Huse JT Ciriello G Poisson LM Barnholtz-Sloan JS Berger MS Comprehensive, integrative genomic analysis of diffuse lower-grade gliomas. N Engl J Med. 2015; 372: 2481–2498. 26061751 10.1056/NEJMoa1402121 PMC4530011

[5] Eckel-Passow JE Lachance DH Molinaro AM Walsh KM Decker PA Sicotte H Pekmezci M Rice T Kosel ML Smirnov IV Sarkar G Caron AA Kollmeyer TM Praska CE Chada AR Halder C Hansen HM McCoy LS Bracci PM Marshall R Glioma groups based on 1p/19q, IDH, and TERT promoter mutations in tumors. N Engl J Med. 2015; 372: 2499–2508. 26061753 10.1056/NEJMoa1407279 PMC4489704

[6] Reifenberger G Hentschel B Felsberg J Schackert G Simon M Schnell O Westphal M Wick W Pietsch T Loeffler M Weller M Predictive impact of MGMT promoter methylation in glioblastoma of the elderly. Int J Cancer. 2012; 131: 1342–1350. 22139906 10.1002/ijc.27385

[7] Reuss DE Sahm F Schrimpf D Wiestler B Capper D Koelsche C Schweizer L Korshunov A Jones DT Hovestadt V Mittelbronn M Schittenhelm J Herold-Mende C Unterberg A Platten M Weller M Wick W Pfister SM von Deimling A ATRX and IDH1-R132H immunohistochemistry with subsequent copy number analysis and IDH sequencing as a basis for an “integrated” diagnostic approach for adult astrocytoma, oligodendroglioma and glioblastoma. Acta Neuropathol. 2015; 129: 133–146. 25427834 10.1007/s00401-014-1370-3

[8] Franco-Hernández C Martínez-Glez V de Campos JM Isla A Vaquero J Gutiérrez M Casartelli C Rey JA Allelic status of 1p and 19q in oligodendrogliomas and glioblastomas: multiplex ligation-dependent probe amplification versus loss of heterozygosity. Cancer Genet Cytogenet. 2009; 190: 93–96. 19380026 10.1016/j.cancergencyto.2008.09.017

[9] Lattanzio L Borgognone M Mocellini C Giordano F Favata E Fasano G Vivenza D Monteverde M Tonissi F Ghiglia A Fillini C Bernucci C Merlano M Lo Nigro C MGMT promoter methylation and glioblastoma: a comparison of analytical methods and of tumor specimens. Int J Biol Markers. 2015; 30: e208–e216. 25588856 10.5301/jbm.5000126

[10] Preusser M Berghoff AS Manzl C Filipits M Weinhäusel A Pulverer W Dieckmann K Widhalm G Wöhrer A Knosp E Marosi C Hainfellner JA Clinical Neuropathology practice news 1-2014: pyrosequencing meets clinical and analytical performance criteria for routine testing of MGMT promoter methylation status in glioblastoma. Clin Neuropathol. 2014; 33: 6–14. 24359605 10.5414/NP300730 PMC3891253

[11] Zacher A Kaulich K Stepanow S Wolter M Kohrer K Felsberg J Malzkorn B Reifenberger G. Molecular diagnostics of gliomas using next generation sequencing of a glioma-tailored gene panel. Brain Pathol. 2016; epub ahead of print. 10.1111/bpa.12367 PMC8029406 26919320

[12] Wiestler B Capper D Sill M Jones DT Hovestadt V Sturm D Koelsche C Bertoni A Schweizer L Korshunov A Weiß EK Schliesser MG Radbruch A Herold-Mende C Roth P Unterberg A Hartmann C Pietsch T Reifenberger G Lichter P Integrated DNA methylation and copy-number profiling identify three clinically and biologically relevant groups of anaplastic glioma. Acta Neuropathol. 2014; 128: 561–571. 25008768 10.1007/s00401-014-1315-x

[13] Andreiuolo F Mazeraud A Chrétien F Pietsch T A global view on the availability of methods and information in the neuropathological diagnostics of CNS tumors: results of an international survey among neuropathological units. Brain Pathol. 2016; 26: 551–554. 27062283 10.1111/bpa.12383 PMC8028915

[14] Aldape K Nejad R Louis DN Zadeh G Integrating molecular markers into the World Health Organization classification of CNS tumors: a survey of the neuro-oncology community. Neuro Oncol. 2016; 10.1093/neuonc/now181 PMC5464323 27688263

[15] Cairncross JG Ueki K Zlatescu MC Lisle DK Finkelstein DM Hammond RR Silver JS Stark PC Macdonald DR Ino Y Ramsay DA Louis DN Specific genetic predictors of chemotherapeutic response and survival in patients with anaplastic oligodendrogliomas. J Natl Cancer Inst. 1998; 90: 1473–1479. 9776413 10.1093/jnci/90.19.1473

[16] Hegi ME Diserens AC Gorlia T Hamou MF de Tribolet N Weller M Kros JM Hainfellner JA Mason W Mariani L Bromberg JE Hau P Mirimanoff RO Cairncross JG Janzer RC Stupp R MGMT gene silencing and benefit from temozolomide in glioblastoma. N Engl J Med. 2005; 352: 997–1003. 15758010 10.1056/NEJMoa043331

[17] Parsons DW Jones S Zhang X Lin JC Leary RJ Angenendt P Mankoo P Carter H Siu IM Gallia GL Olivi A McLendon R Rasheed BA Keir S Nikolskaya T Nikolsky Y Busam DA Tekleab H Diaz LA Hartigan J An integrated genomic analysis of human glioblastoma multiforme. Science. 2008; 321: 1807–1812. 18772396 10.1126/science.1164382 PMC2820389

[18] Rennert H Eng K Zhang T Tan A Xiang J Romanel A Kim R Tam W Liu Y-C Bhinder B Cyrta J Beltran H Robinson B Mosquera JM Fernandes H Demichelis F Sboner A Kluk M Rubin MA Elemento O Development and validation of a whole-exome sequencing test for simultaneous detection of point mutations, indels and copy-number alterations for precision cancer care. Npj Genomic Medicine.. 2016; 1: 16019. 10.1038/npjgenmed.2016.19 PMC5539963 28781886

[19] Sahm F Schrimpf D Jones DT Meyer J Kratz A Reuss D Capper D Koelsche C Korshunov A Wiestler B Buchhalter I Milde T Selt F Sturm D Kool M Hummel M Bewerunge-Hudler M Mawrin C Schüller U Jungk C Next-generation sequencing in routine brain tumor diagnostics enables an integrated diagnosis and identifies actionable targets. Acta Neuropathol. 2016; 131: 903–910. 26671409 10.1007/s00401-015-1519-8

[20] Nikiforova MN Wald AI Melan MA Roy S Zhong S Hamilton RL Lieberman FS Drappatz J Amankulor NM Pollack IF Nikiforov YE Horbinski C Targeted next-generation sequencing panel (GlioSeq) provides comprehensive genetic profiling of central nervous system tumors. Neuro-oncol. 2016; 18: 379–387. 26681766 10.1093/neuonc/nov289 PMC4767245

[21] Blumenthal DT Dvir A Lossos A Tzuk-Shina T Lior T Limon D Yust-Katz S Lokiec A Ram Z Ross JS Ali SM Yair R Soussan-Gutman L Bokstein F Clinical utility and treatment outcome of comprehensive genomic profiling in high grade glioma patients. J Neurooncol. 2016; 130: 211–219. 27531351 10.1007/s11060-016-2237-3

[22] Green RC Berg JS Grody WW Kalia SS Korf BR Martin CL McGuire AL Nussbaum RL O’Daniel JM Ormond KE Rehm HL Watson MS Williams MS Biesecker LG ACMG recommendations for reporting of incidental findings in clinical exome and genome sequencing. Genet Med. 2013; 15: 565–574. 23788249 10.1038/gim.2013.73 PMC3727274

